# Advantages of Using Fibres to Withstand Shear Stress: A Comparative Parametric Study with Conventionally Reinforced Concrete Beams

**DOI:** 10.3390/ma18040801

**Published:** 2025-02-12

**Authors:** Alvaro Picazo, Marcos García Alberti, Alejandro Enfedaque, Jaime C. Gálvez

**Affiliations:** 1 Departamento de Tecnología de la Edificación, E.T.S. de Edificación, Universidad Politécnica de Madrid, Avda. Juan de Herrera, 6, 28040 Madrid, Spain; a.picazo@upm.es; 2 Departamento de Ingeniería Civil: Construcción, E.T.S. de Ingenieros de Caminos, Canales y Puertos, Universidad Politécnica de Madrid, C/Profesor Aranguren, s/n, 28040 Madrid, Spain; alejandro.enfedaque@upm.es (A.E.); jaime.galvez@upm.es (J.C.G.)

**Keywords:** fibre reinforced concrete, steel fibre, polyolefin fibre, shear, shear reinforcement

## Abstract

The structural use of fibre-reinforced concrete (FRC) has shown to be an attractive alternative for certain structural elements, being especially suitable to withstand shear stresses in concrete beams. In the case of longitudinal steel bars to support bending stresses, the reductions are of interest. However, in the case of shear stress, it is possible to eliminate the stirrup reinforcement in certain areas. In such a case, the use of FRC may eliminate not only the material but also the tasks of preparing and placing reinforcement, achieving significant savings in labour and allowing a faster execution, avoiding human error, and providing greater security to the work. This study was developed with the aim of assessing a basic practical application of FRC for shear strength. A series of graphics have been made to be used as a calculation tool. The typical structural elements of buildings subjected to bending and shear stress have been tested and analysed. The results for steel fibre-reinforced concrete (SFRC) and polyolefin fibre-reinforced concrete (PFRC) show that fibre can substitute, to some extent, part of the longitudinal reinforcement needed to provide the required flexural strength. Additionally, the fibres can reduce or even eliminate the need for stirrups for shear strength, which leads to savings in cost and execution time.

## 1. Introduction

The widespread use of concrete makes it the most widely used structural material in the fields of both civil engineering and building construction. This extensive use promotes the study of the material as a resistant element to optimize its use, seeking the use of smaller sections, a consequent reduction in the structure weight, and a reduction in environmental impacts [1]. The design of smaller section pieces, on the one hand, requires an increase in the concrete compressive strength and, on the other hand, demands a reinforcement material be supplied in areas where the concrete cannot withstand the stress (tension areas). Concrete is, in itself, a material with very good compression behaviour [2], but at the same time, it is an almost fragile material [3] with a low tension strength. For these reasons, concrete must be reinforced to be used structurally. There are different types of reinforcements that can be combined with concrete in order to achieve the required tension strength and ductility in a structural member.

The most used method consists of embedding steel bars in the concrete mass. This solution, called reinforced concrete (RC), requires the placement of the bars in certain positions so that the calculated internal forces can be resisted, either complementing the concrete compression strength or providing the steel tension strength.

This technique is considered standard, and there are regulations governing its use [4,5,6]. To make this type of reinforcement, the work of cutting, bending, placing, and tying the bars is necessary [7], a process that is still carried out practically by hand. This means that a significant part of an RC unit’s labour cost is comprised of its rebar labour [8]. Another type of reinforcement consists of adding randomly arranged fibres into a concrete mass. This material is known as fibre-reinforced concrete (FRC) and has experienced significant development since the middle of the last century [9]. The current codes of structural design [4,5,10] contemplate the way in which the inclusion of reinforcement fibres can be considered in structural calculations, taking as a base the use of steel fibres, that is, steel fibre-reinforced concrete (SFRC). In recent years, the chemical industry has developed another type of structural reinforcement fibre. They are polymeric fibres suitable to be used with structural character [11] and with chemical stability properties that improve the problem of corrosion [12]. The reinforcement with this type of fibre is known as polyolefin fibre-reinforced concrete (PFRC).

In both cases, the fibres are added during the concrete mixing, requiring no additional machinery. Likewise, no special means are required for concreting, similar to plain concrete. In addition to the reduction in the placing costs of FRC, it is worth mentioning two advantages: it avoids concrete short-age shrinkage cracking and limits concrete cracking in the service state of the structure [13]. The presence of fibres provides a bridging effect in the concrete mass, resulting in multi-cracking with smaller cracks than those produced in concrete without fibres. Bigger cracks are prone to increase in size, both in length and opening [14].

The structural contribution of the fibres means that for certain stress conditions, the steel bar reinforcement can be reduced, or may even become unnecessary [15]. In beams, longitudinal steel bars are necessary to support bending stresses [16], but in the case of shear stress, it is possible to eliminate the stirrup reinforcement. In this case, the use of FRC removes the tasks of preparing and placing reinforcement, achieving significant savings in labour and having faster execution, avoiding human error, and providing greater security to the work. The use of FRC has been successful in tunnel linings [17], pipelines [18,19], foundation slabs [20], road bridges [21], and building roofs [22].

The practical use of FRC to achieve cost savings requires complying with the design criteria ruled by the standards [4,5]. The strength criteria in the codes are currently based on the residual bending tensile stress values [23]. Some authors have demonstrated that this is not the correct way to consider the contribution of fibre reinforcement to shear stress [24]. However, in the absence of a possible modification of the codes, this study is based on the residual FRC strength as indicated by the current rules.

Under this premise, this study was developed with the aim of exploring a basic practical application of FRC for shear strength. A series of graphics were made to be used as a calculation tool. The typical structural elements of buildings subjected to bending and shear stress were tested and analysed. These elements were studied with different forms and load values, using FRC as a resistant material with different volumetric fibre fractions, and then compared to those fabricated with RC. The results demonstrate the good behaviour of the fibre reinforcement towards shear forces while showing significant savings in structure construction in terms of time and cost. Also, the element fabrication with FRC is less dependent on labour and avoids possible human errors while showing an improvement in execution safety.

The parametric analysis that carried out in this study provides designers with an easy and reliable way of determining whether the steel stirrups that are employed to sustain shear stresses could be substituted by a fibre-reinforced concrete. The most relevant change that the application of these findings may bring to any construction site is a reduction in the labour force required in the structure construction stage. Moreover, the presence of fibres would introduce a homogeneous dispersed reinforcement that might not only reduce the width of cracks that can appear in the structural elements but may also increase the life span of the structure. The latter would be a consequence of the decrease in the ingress rate of potential deleterious substances through the cracks and would also reduce the environmental impact of the structure.

## 2. Assumptions and Starting Data

When addressing a practical study of the use of FRC and the possible reduction or elimination of shear bar reinforcement, different approaches, methodologies, and variables can be considered.

Due to the enormous diversity of options and considering that the purpose of this study was not the production of an FRC calculation program but rather a comparative study between FRC and RC with traditional shear reinforcement, the following considerations and data were assumed.

Among the different standards that regulate the calculation of structural concrete, the new Spanish Structural Code CE-21 [5] was chosen because its territorial scope coincides with this study, and the formulation is similar to that of the Model Code CM-2020 [4]. In previous research, a comparison was made between the shear calculation made by the old Spanish Structural Code EHE-08, similar to CE-21 and the CM-2020, showing slight differences [25].

Regarding the structural elements, rectangular section beams with the usual building dimensions were selected. The spans were 2000, 4000 and 6000 mm. The total height of the beams varied between 300 and 500 mm, always with a design cover thickness of *d’* = 40 mm, and the widths ranged between 250 and 300 mm.

**Figure 1:**
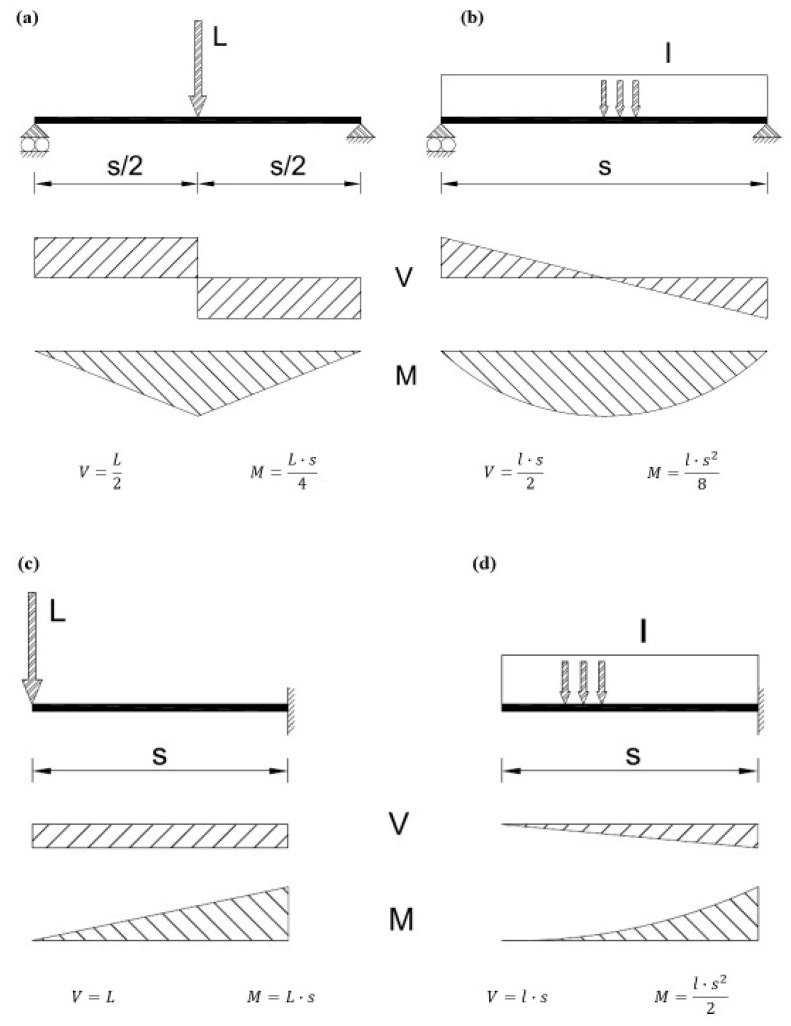
Structural loading schemes and shear and bending diagrams: (**a**) simply supported beam with point load at mid-length, (**b**) simply supported beam with uniformly distributed load, (**c**) cantilever beam with point load, and (**d**) cantilever beam with the uniformly distributed load.

With respect to the structural configuration, a simply supported beam was considered, with two types of loadings: a central point load and a uniformly distributed load over the entire length of the beam. Also, cantilever beams with point load and uniformly distributed load were studied. The structural schemes of the beams, along with their shear and bending moment diagrams, are shown in Figure 1.

The applied load values included both permanent and variable loads that are typical in building standards: floor supporting system, flooring, hanging loads, partition walls, and variable use load. Thus, uniformly distributed loads of 4 kN/m^2^ and 2 kN/m^2^ were considered as minimum values for the permanent and variable loads, respectively. Load increments were always a 5% increase.

The materials used were as follows: self-compacting concrete with compression strength *f_ck_* = 25 MPa and modulus of elasticity *E_c_* = 30,000 MPa; steel bars with elastic limit *f_y_* = 500 MPa and modulus of elasticity *E_s_* = 210,000 MPa. The safety coefficients of the load and material strengths were those for ultimate limit state calculation according to CE-21 [5]. It has been demonstrated that fibres can have structural character if they meet certain conditions [4,5] based on bending residual tensile strengths. Research has shown that the minimum amounts of fibres included in concrete that provide structural character are 20 kg/m^3^ in the case of steel fibres, and 5 kg/m^3^ in the case of polyolefin fibres. For this reason, in this study, reinforcements of 20, 35, 50, and 70 kg/m^3^ of steel fibres and 6 and 10 kg/m^3^ of polyolefin fibres were used. The bar diameters used for the reinforcement were *ø* 16 mm for the longitudinal reinforcement, and *ø* 8 mm for stirrups. Table 1 summarizes the dimensions of the beams and the types of FRC used, Table 2 shows the main characteristics of the fibres, and Table 3 shows the mix dosage concrete and the process of making concrete.

In accordance with EN-14651 [23], three-point bending tests were carried out in prismatic specimens. For an accurate positioning of the specimen on the support points and the location of the load, a laser level was used. The test was initially controlled by a clip-on gage crack mouth opening displacement (CMOD) device placed on a central notch. Deflection was also measured by two linear variable differential transformer (LVDT) devices on each side of the specimen. Time, load, and the machine actuator position data were also registered. When FRC deformations were higher than the upper limit of the CMOD device, the test control was changed to the actuator position until the end of the test.

**Figure 2:**
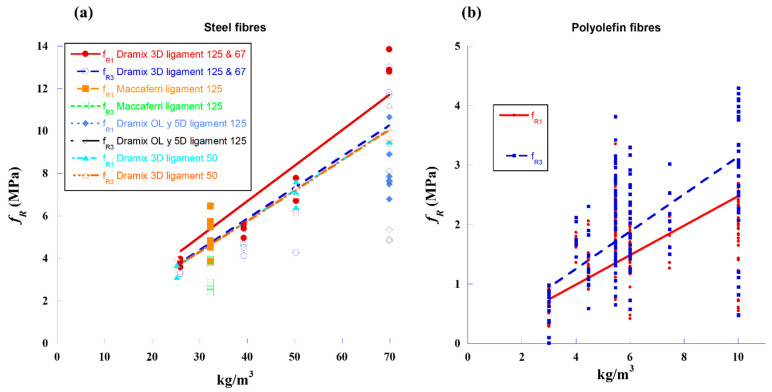
Residual strength values *f_R1_* and *f_R3_* vs. fibre reinforcement content: (**a**) steel fibres and (**b**) polyolefin fibres.

In the FRC testing, the residual bending tensile strengths (*f_R3_*) were recorded for a CMOD value of 2.5 mm, obtained by three-point bending tests according to EN-14651 [23]. These data were obtained from the results of 190 previous tests [26,27], on which Figure 2 was based. Figure 2a shows the relations between the residual strengths *f_R1_* and *f_R3_* versus the amount of steel fibres. Similarly, Figure 2b relates *f_R1_* and *f_R3_* to the amount of polyolefin fibres. These data are necessary to relate the residual strengths with the volumetric fraction and the number of fibres (steel or polyolefin), which allowed for consideration of the different strength values for further calculations. According to EN-14651 [23], *f_R1_* (associated with the serviceability limit state) is the residual bending tensile strength at CMOD 0.5 mm, and *f_R3_* (associated with the ultimate limit state) is the residual bending tensile strength at CMOD 2.5 mm. These values, compared as a percentage with the strength at the proportionality limit (*f_LOP_*), mark the possibility of considering the structural behaviour of the fibres if *f_R1_*/*f_LOP_* > 40% and *f_R3_*/*f_LOP_* > 20% are met.

In the case of the reinforcement with steel fibres, Dramix^®^ 3D steel fibres were used. According to Figure 2a, for these fibres, and assuming a linear relation between *f_R3_* and the fibre dosage *ρ*, the relation can be expressed as *f_R3_* (MPa) = 0.1468· *ρ* (kg/m^3^). Therefore, to obtain a value of 3.0 MPa for *f_R3_*, it is necessary to use *ρ* = 20.44 kg/m^3^ of steel fibres.

Regarding the addition of polyolefin fibres, *f_R3_* can be expressed in relation to the fibre dosage as follows: *f_R3_* (MPa) = 0.3144 *ρ* (kg/m^3^) where *ρ* is the amount of fibre added to the concrete mix. Therefore, for obtaining a residual strength at CMOD equal to 2.5 mm equal to 1.5 MPa, a fibre dosage of *ρ* = 4.77 kg/m^3^ is required.

As a simplification, it has been assumed that the longitudinal tension and compression reinforcements obtained for the maximum bending moment are arranged continuously along the entire element length. The calculation was performed for the maximum shear design value.

The shear reinforcement calculation of RC beam considered rectangular stirrups at 90° to the piece longitudinal direction. For the rest of the calculations, the required reinforcement area was assessed in cm^2^, without converting it to a real number of bars.

In addition to the savings in execution time produced by the replacement or elimination of stirrups by the use of FRC, the cost aspects are significant. As mentioned above, one of the advantages of FRC over traditional reinforcement is the substantial reduction in labour, avoiding errors in reinforcement assembling and positioning. In order to carry out a cost comparison of the RC with the FRC, the work unit price generator for Spain© developed by Cype Ingenieros, S.A. was used on the ninth day of October 2024 [8], as reviewed by IECA (Spanish Institute of Cement and its Applications), taking the prices shown in Table 4. Self-compacting concrete with 25 N/mm^2^ compression strength was considered, both for RC and FRC, with a 12 mm maximum aggregate size and located in environment Type X0.

The assessment of the amount of steel reinforcement as per Table 4 [8] allowed for the evaluation of labour time savings achieved when using FRC instead of RC. According to the unit price break down per each additional kg of reinforcing steel, 0.022 man-hours are required, with a cost of EUR 0.43. The material used in the unit price amounts to 1.64 EUR/kg.

The longitudinal tensile reinforcement was assessed using the CE-21 [5] formulas, whereas the following formulas were used for the checking to shear strength: Equation (1) for the calculation of the total shear strength, Equation (2) to check that the oblique compression struts do not fail, and Equation (3) to verify that the minimum shear strength due to stirrups and fibres is met. In some cases, the analytical results assumed the presence of a minimum number of stirrups when required by the code. In all cases, the results of the study considered the contribution of both fibres and stirrups.

(1) $$V_{u2}=V_{cu}+V_{su}+V_{fu}$$

(2) $$V_{Rd,max}=\alpha_{cw}\cdot b_{w}\cdot z\cdot \upsilon_{1}\cdot f_{cd}/\left(\cot\theta +\tan\theta \right)$$

(3) $$V_{su}+V_{fu}>\frac{f_{ct,m}}{7.5}{\cdot b}_{0}\cdot d$$

where *V_u2_* is the shear resistance of the web due to tension failure;

*V_cu_* is the contribution of concrete to shear resistance;

*V_su_* is the contribution of steel reinforcement to shear resistance;

*V_fu_* is the contribution of the fibres to shear resistance;

*V_Rd,max_* is the design shear resistance;

*α_cw_* is the reduction coefficient for considering compressive stress field;

*b_w_* is the minimum width between tension and compression areas;

*z* is the internal lever arm;

*υ*_1_ is the reduction coefficient for considering the cracking of concrete;

*f_cd_* is the design compressive strength of concrete;

*θ* is the inclination of the compressive stress field;

*b*_0_ is the width of the web;

*d* is the effective depth to main tension reinforcement;

*f_ct_*_,m_ is the average tension strength of concrete.

## 3. Results

The FRC weight changes depending on the type of fibre reinforcement, steel or polyolefin fibres, used. For this reason, considering that for the same value of the residual strength *f_R3_* the amount of fibre depends on the material type, the results have been organized separately for the SFRC and PFRC. Also, in order to focus on relevant results, some representative graphs have been selected out of a total generated casuistry of 150 graphs.

The purpose of the analysis was not to compare the analytical results between the different types of beams, but to look for graphs that would make it possible to clearly assess the material amounts, costs, and construction time. The values of the latter parameters are shown in the abscissa axis of the following graphs, generally as a function of the applied design load for different beam configurations.

### 3.1. Results for Steel Fibre-Reinforced Concrete

**Figure 3:**
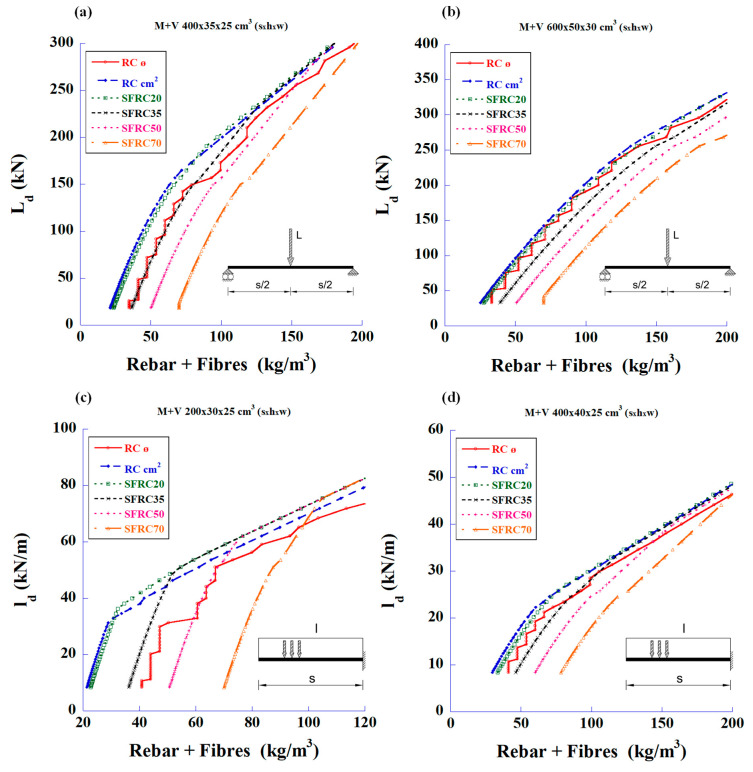
Maximum design values of the applied load vs. total amount of steel to meet the required bending and shear strengths: (**a**) 4 m-long simply supported beam with mid-span concentrated load, (**b**) 6 m-long simply supported beam with mid-span concentrated load, (**c**) 2 m-long cantilever beam with uniformly distributed load, and (**d**) 4 m-long cantilever beam with uniformly distributed load.

With the analysis results, the graphs in Figure 3 were plotted to show the total steel quantities (steel bars plus fibre weight) versus the maximum applied load design values.

Initially, it can be observed that with fibre values of 20 kg/m^3^, the beams support the same load as the rebar solution, but with a lower total steel weight. With 35 kg/m^3^ of fibres, both solutions are close, occasionally with a lower total weight for one or the other. Finally, the FRC with 50 and 70 kg/m^3^ fibre contents showed that they should be reserved for high loads.

**Figure 4:**
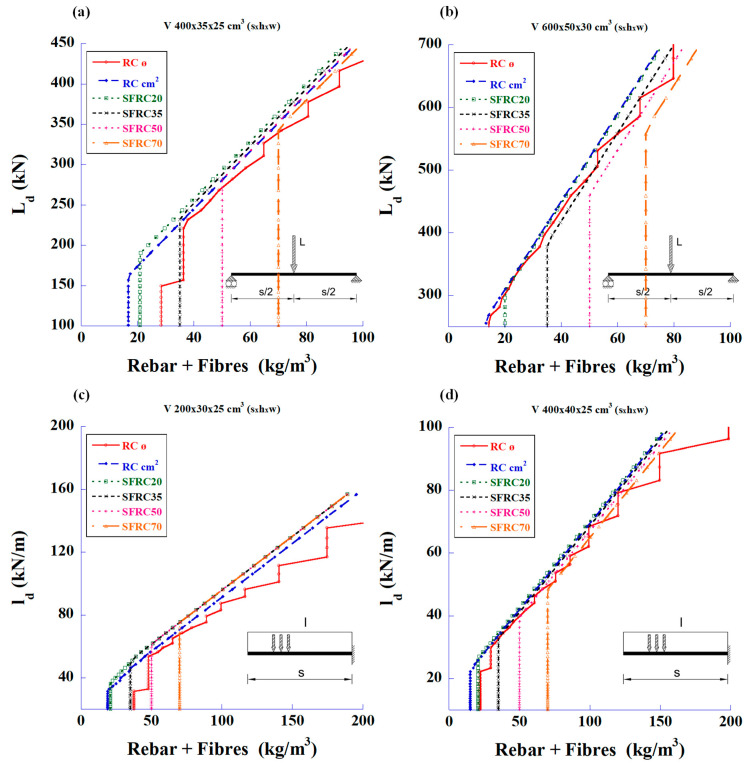
Maximum design values of the applied load vs. total amount of steel to meet only the required shear strength: (**a**) 4 m-long simply supported beam with mid-span concentrated load, (**b**) 6 m-long simply supported beam with mid-span concentrated load, (**c**) 2 m-long cantilever beam with uniformly distributed load, and (**d**) 4 m-long cantilever beam with uniformly distributed load.

The graphs in Figure 4 show the calculations based only on the shear strength for the same types of beams and loads. The steel amount is the sum of the rebar plus fibre content.

**Figure 5:**
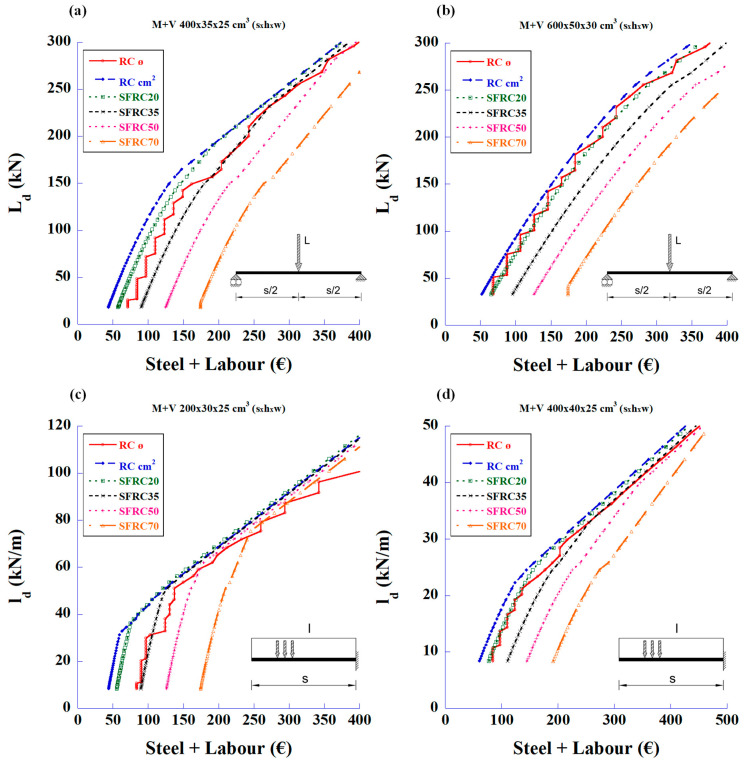
Maximum design values of the applied load vs. reinforcement price of total amount of steel and labour to meet the required bending and shear strengths: (**a**) 4 m-long simply supported beam with mid-span concentrated load, (**b**) 6 m-long simply supported beam with mid-span concentrated load, (**c**) 2 m-long cantilever beam with uniformly distributed load, and (**d**) 4 m-long cantilever beam with uniformly distributed load.

The first statement that can be deduced from these figures is that the fibres can meet the required shear strength without the need for traditional stirrups. The second parameter to be considered is the cost of the structure associated with the prices of materials and labour. Figure 5 shows the relationship between the design loads and the total reinforcement prices of total steel and labour for the bending and shear strengths.

In Figure 5, it can be seen that the SFRC with a high amount of fibre content (50 and 70 kg/m^3^) is only economical compared to the RC in the 2 m-long cantilever beams.

**Figure 6:**
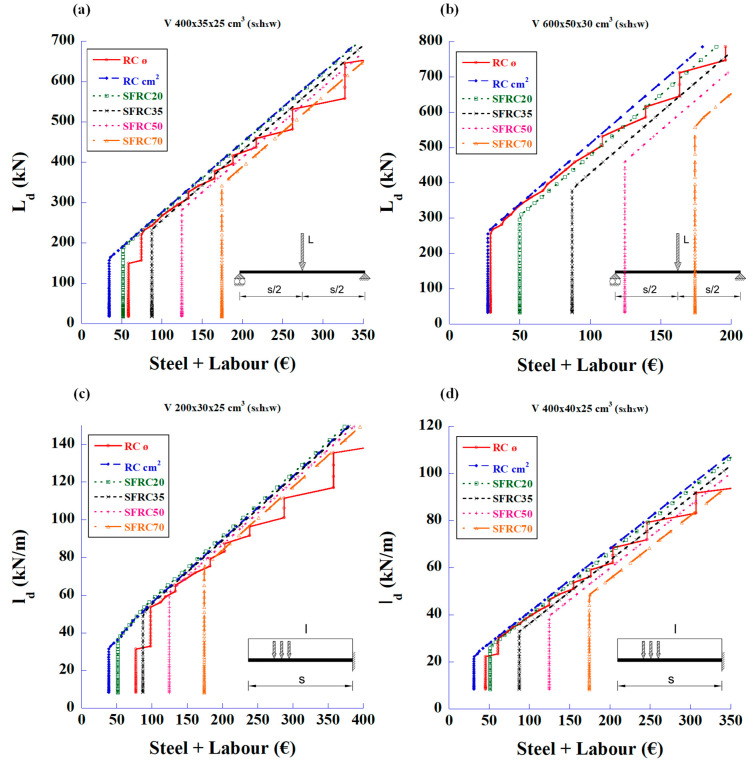
Maximum design values of the applied load vs. reinforcement price of total amount of steel and labour to meet only the required shear strength: (**a**) 4 m-long simply supported beam with mid-span concentrated load, (**b**) 6 m-long simply supported beam with mid-span concentrated load, (**c**) 2 m-long cantilever beam with uniformly distributed load, and (**d**) 4 m-long cantilever beam with uniformly distributed load.

The graphs shown in Figure 6 relate the cost of materials and labour with the design load for shear strength only. It can be seen that, as in Figure 5, the SRFC with 50 or 70 kg/m^3^ of fibre content is not economical when replacing the traditional stirrups, except in cantilever beams of moderate spans.

The execution time spent on the construction of a structure is also significant. In fact, it can be the more important factor when choosing FRC instead of the traditional steel bar reinforcement. The following sets of graphs have been developed in consideration of this factor.

**Figure 7:**
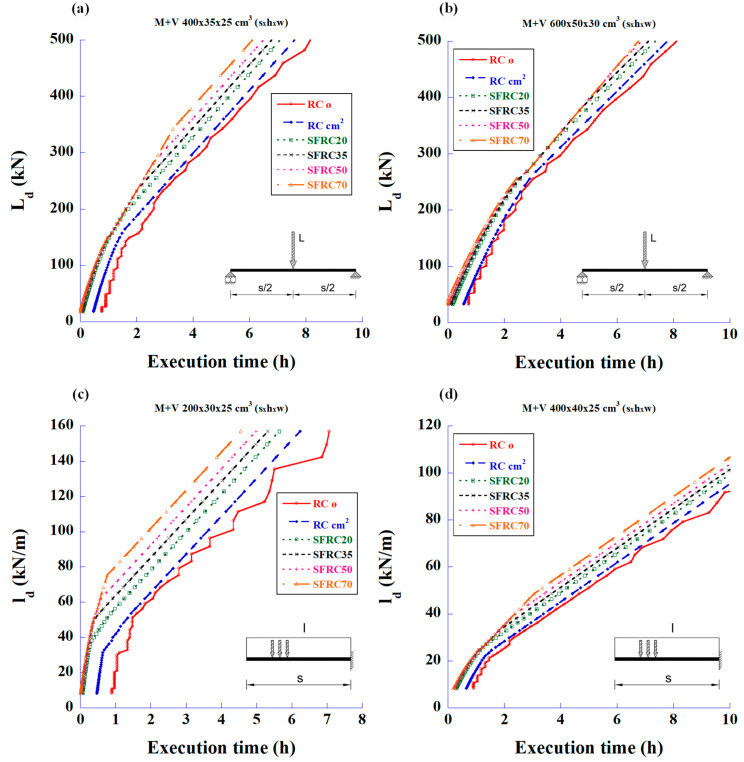
Maximum design values of the applied load versus working time spent in reinforcing for bending and shear using RC and SFRC: (**a**) 4 m-long simply supported beam with mid-span concentrated load, (**b**) 6 m-long simply supported beam with mid-span concentrated load, (**c**) 2 m-long cantilever beam with uniformly distributed load, and (**d**) 4 m-long cantilever beam with uniformly distributed load.

Figure 7 shows the relationship between the maximum design loads versus work time spent in reinforcing two types of beams. This time is the sum of the work time (cutting, bending, and cage assembling) of the longitudinal bending reinforcements and stirrups. In these graphs, it can be clearly seen that the SFRC with the highest amount of fibres (70 kg/m^3^) always shows the shortest execution time for any load.

**Figure 8:**
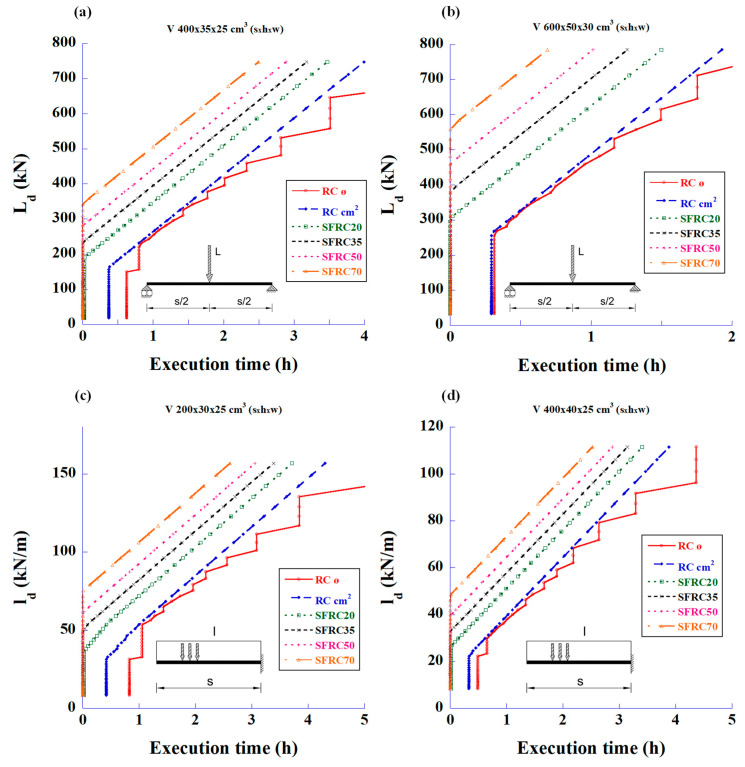
Maximum design values of the applied load versus working time spent in reinforcing for shear using RC and SFRC: (**a**) 4 m-long simply supported beam with mid-span concentrated load, (**b**) 6 m-long simply supported beam with mid-span concentrated load, (**c**) 2 m-long cantilever beam with uniformly distributed load, and (**d**) 4 m-long cantilever beam with uniformly distributed load.

The graphs in Figure 8 show the relationship between the maximum design loads and execution time taken to provide the reinforcement to resist shear forces. These graphs are particularly interesting since they mark the load limit at which the fibres can completely replace the stirrups. The SFRC70 covers the stirrup capacity up to a load of 343 kN, the SFRC50 up to 268 kN, and the SFRC35 up to 232 kN. The rest of the SFRC cases need minimum amounts of stirrups to comply with CE-21, according to Equation (3).

### 3.2. Results for Polyolefin Fibre-Reinforced Concrete

**Figure 9:**
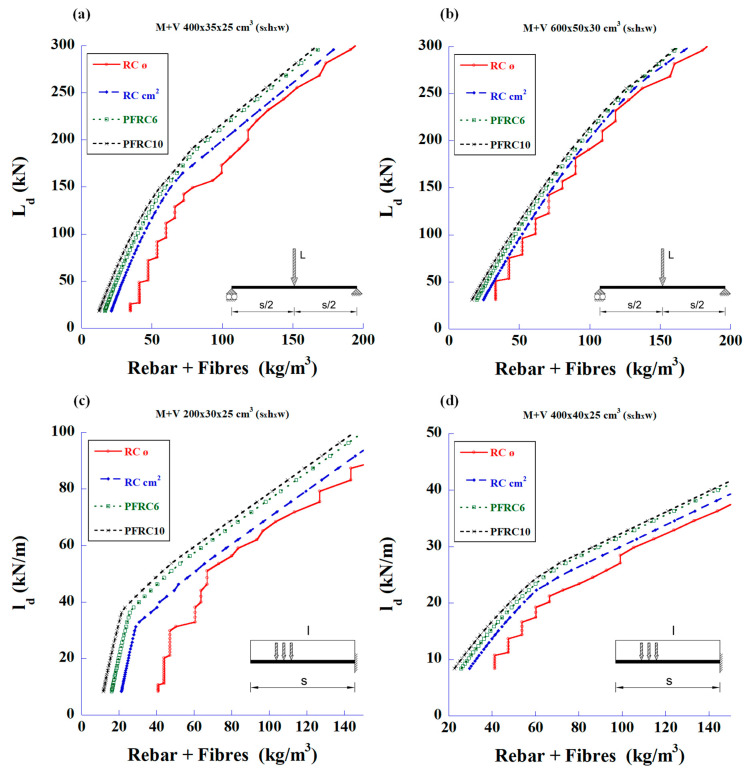
Maximum design values of the applied load vs. total weight of reinforcing steel and polyolefin fibres to meet the required bending and shear strengths: (**a**) 4 m-long simply supported beam with mid-span concentrated load, (**b**) 6 m-long simply supported beam with mid-span concentrated load, (**c**) 2 m-long cantilever beam with uniformly distributed load, and (**d**) 4 m-long cantilever beam with uniformly distributed load.

In Figure 9, graphs relating the maximum design load to the amount of the reinforcing materials are shown. The amount is the sum of polyolefin fibres and steel in traditional bar reinforcement required to meet the necessary bending and shear strengths in the analysed element.

It becomes evident that in all cases, the required reinforcing material weight is always smaller for the PFRC as compared to reinforcement with steel bars, especially for the shorter beams.

**Figure 10:**
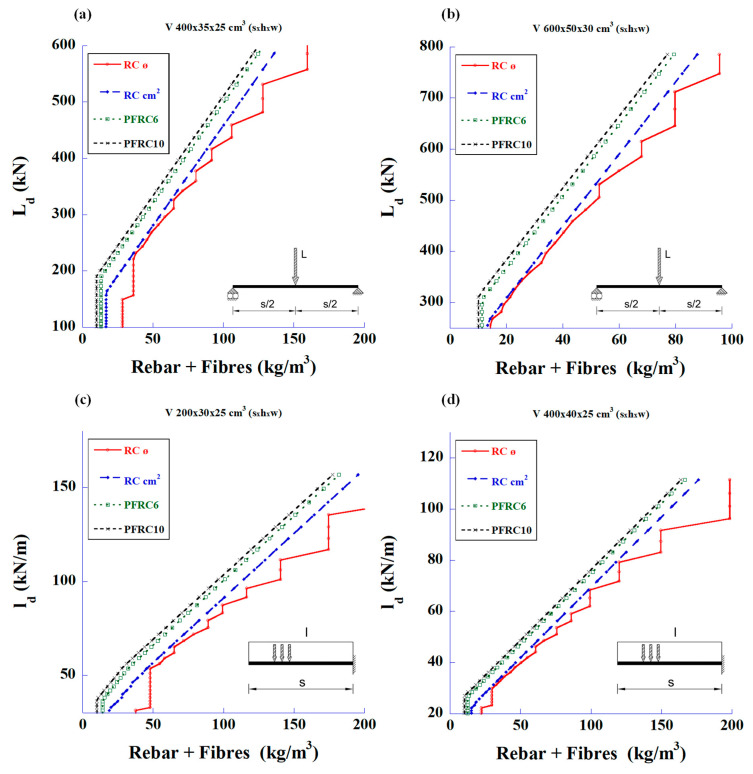
Maximum design values of the applied load vs. total weight of steel stirrups and polyolefin fibres to meet only the required shear strength: (**a**) 4 m-long simply supported beam with mid-span concentrated load, (**b**) 6 m-long simply supported beam with mid-span concentrated load, (**c**) 2 m-long cantilever beam with uniformly distributed load, and (**d**) 4 m-long cantilever beam with uniformly distributed load.

Figure 10 shows the graphs for the case of designing to resist the shear forces. The total weight now is the sum of the stirrups and the polyolefin fibre weights.

It can be observed in Figure 10 that the fibres alone are able to withstand the shear forces without any other steel reinforcement, up to certain load values. This is more pronounced in the simply supported beams as compared to the cantilever beams.

**Figure 11:**
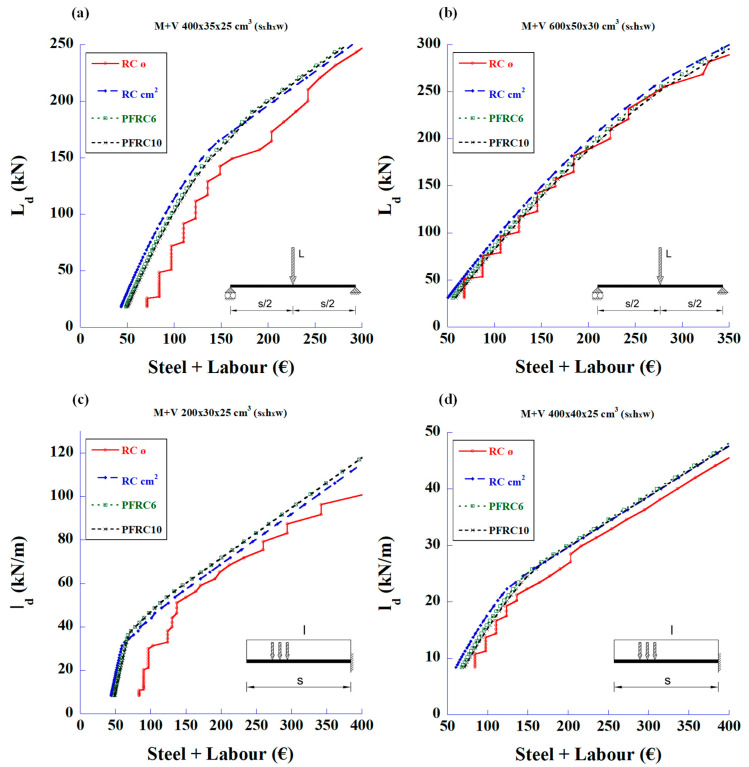
Maximum design values of the applied load vs. reinforcement price of total amount of steel, labour and polyolefin fibres to meet the required bending and shear strengths using either RC or PFRC: (**a**) 4 m-long simply supported beam with mid-span concentrated load, (**b**) 6 m-long simply supported beam with mid-span concentrated load, (**c**) 2 m-long cantilever beam with uniformly distributed load, and (**d**) 4 m-long cantilever beam with uniformly distributed load.

Figure 11 was prepared to show the relationship between the maximum design loads and work unit costs (materials plus labour) required to meet the strength requirements of bending and shear using steel bars and polyolefin fibres.

In Figure 11 clearly shows that PFRC is more economical than RC, especially in the shorter beams.

**Figure 12:**
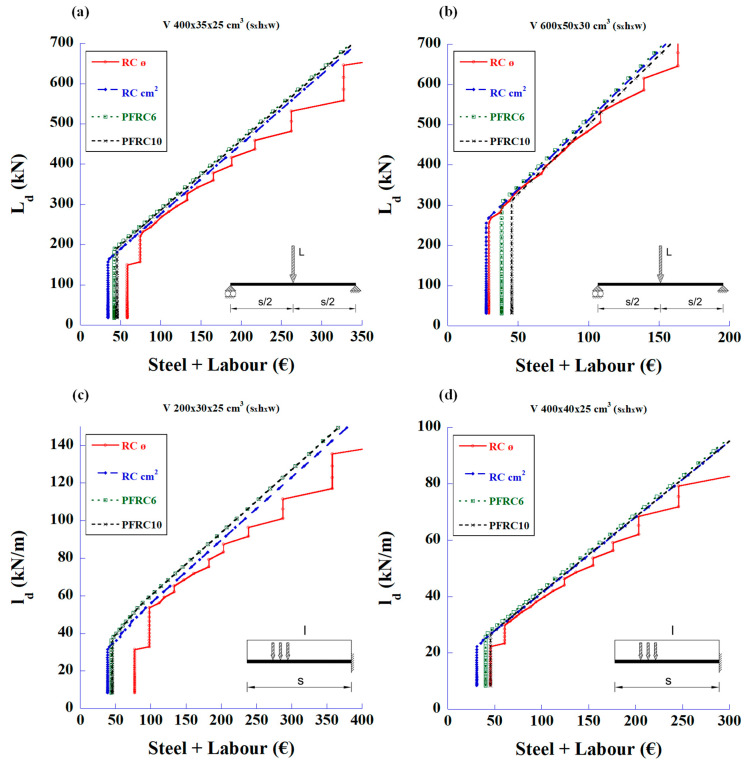
Maximum design values of the applied load vs. work unit price of total of steel, labour and polyolefin fibres to meet the required shear strength using either RC (stirrups) or PFRC: (**a**) 4 m-long simply supported beam with mid-span concentrated load, (**b**) 6 m-long simply supported beam with mid-span concentrated load, (**c**) 2 m-long cantilever beam with uniformly distributed load, and (**d**) 4 m-long cantilever beam with uniformly distributed load.

The graphs in Figure 12 were obtained by considering only the shear strength in the design. Again, the conclusions are similar to the those in Figure 11.

**Figure 13:**
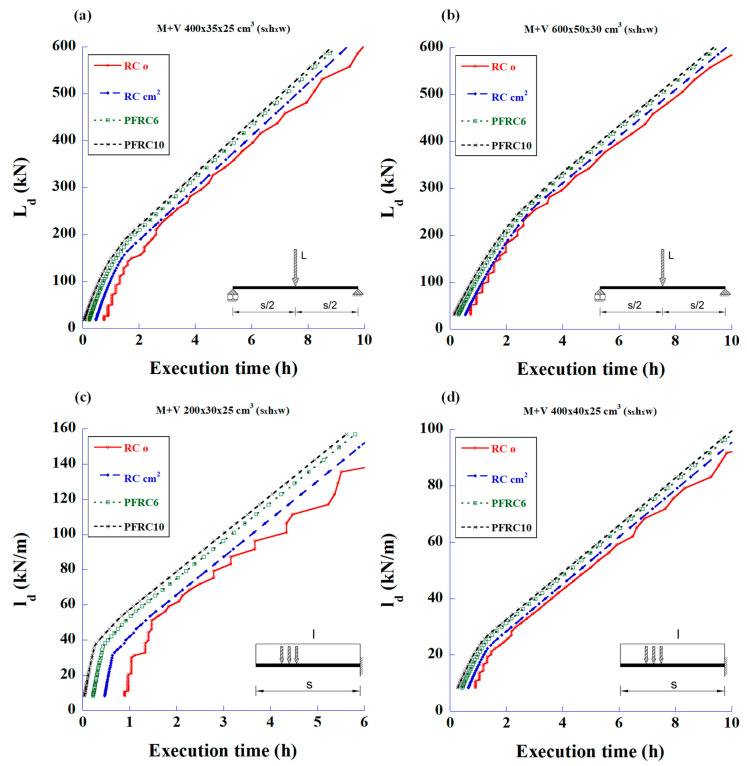
Maximum design values of the applied load vs. execution time to perform all activities for the steel reinforcement and meet the required bending and shear strengths using either RC or PFRC: (**a**) 4 m-long simply supported beam with mid-span concentrated load, (**b**) 6 m-long simply supported beam with mid-span concentrated load, (**c**) 2 m-long cantilever beam with uniformly distributed load, and (**d**) 4 m-long cantilever beam with uniformly distributed load.

Execution time graphs, similar to the ones for SFRC, were prepared, as shown in Figure 13, where time refers to the sum of the time required for the bar cutting, bending, assembling, and placing of longitudinal reinforcement and stirrups. PFRC10 shows a shorter execution time for any load value.

**Figure 14:**
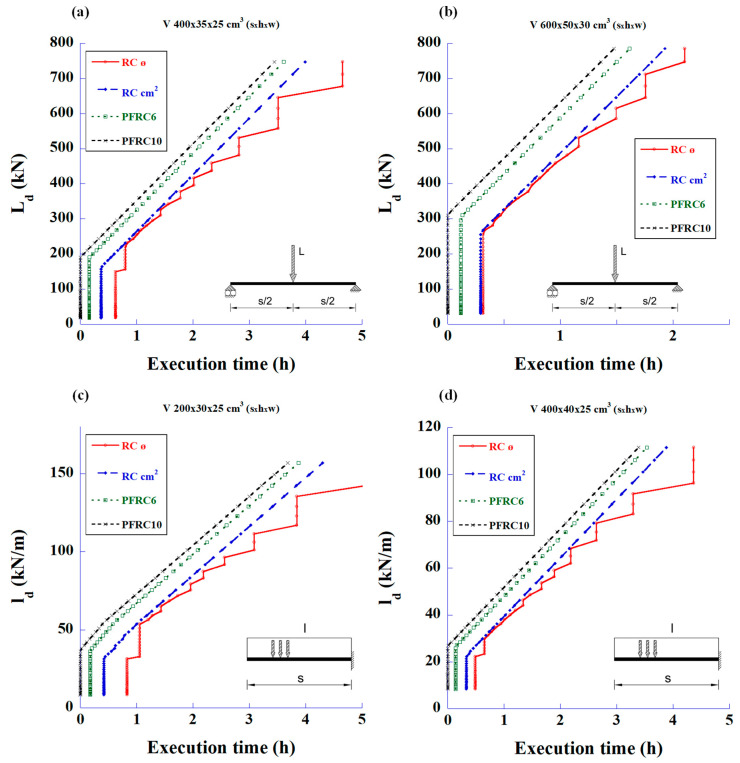
Maximum design values of the applied load vs. execution time to perform all activities for the steel stirrups and/or polyolefin fibres and meet the required shear strength using either RC or PFRC: (**a**) 4 m-long simply supported beam with mid-span concentrated load, (**b**) 6 m-long simply supported beam with mid-span concentrated load, (**c**) 2 m-long cantilever beam with uniformly distributed load, and (**d**) 4 m-long cantilever beam with uniformly distributed load.

Figure 14 shows the relationship between the maximum design loads vs. execution time, but for meeting the required shear strength only. The importance of these graphs relies upon the fact that they demonstrate that the stirrups could be eliminated with the use of polyolefin fibres. Thus, in Figure 14a, the fibres of PFRC10 cover the required strength up to a design load of 191 kN, and in Figure 14b up to a design load of 311 kN. It can also be observed that PFRC6 cannot completely substitute the stirrups due to the minimum conditions ruled by Equation (3) of CE-21.

All the above graphs demonstrate that the use of fibres to reduce or substitute bar reinforcement is a good, practical solution. Fibres can reduce a small portion of the bending bar reinforcement while maintaining a high efficiency in meeting the shear strength and, in some cases, they can completely replace the need for stirrups, as shown in more detail below.

## 4. Analysis of Results and Replacement of Stirrups

The following discussions are based on the results obtained for mixes SFRC20, SFRC35, PFRC6, and PFRC10 for shear reinforcement. Mixes SFRC50 and SFRC70 came close to achieving stirrup replacement but were more expensive and so are not included in the discussion.

### 4.1. Analysis of Results

The analysis aimed to select the parameters that most influence the decision to use fibres to improve the beam execution while meeting the required shear strength. The beam length was considered a main parameter, with the section dimensions kept constant. Distributed loads were also selected because they are more frequent in building construction compared with concentrated loads. The fibre-reinforced mixes analysed were PFRC with 6 and 10 kg/m^3^ of polyolefin fibres and SFRC with 20 and 35 kg/m^3^ of steel fibres. Under these premises, new comparative graphs were prepared.

**Figure 15:**
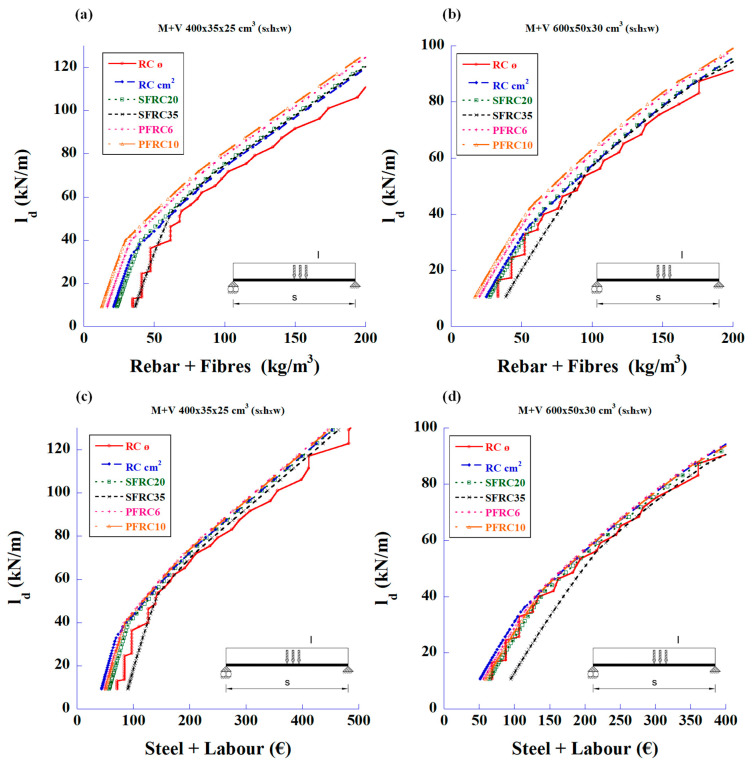

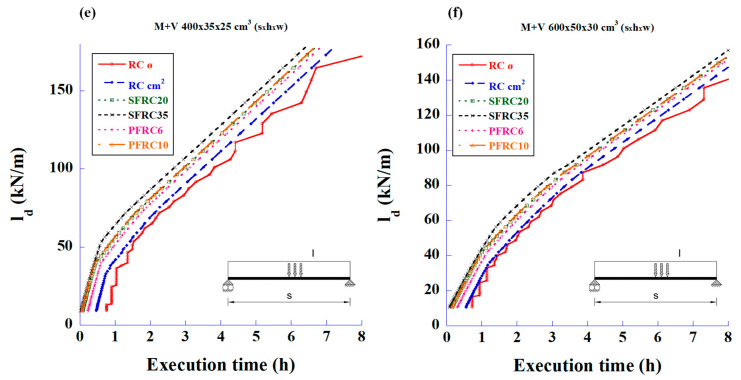
Simply supported beams with uniform load reinforced for bending and shear. Graphs relating design load vs. reinforcing material weight; design load vs. cost of reinforcing materials including labour; and design load vs. execution time: (**a**) load–weight for 4 m-long beam, (**b**) load–weight for 6 m-long beam, (**c**) load–cost for 4 m-long beam, (**d**) load–cost for 6 m-long beam, (**e**) load–time for 4 m-long beam, and (**f**) load–time for 6 m-long beam.

Figure 15 shows the comparison for two simply supported, 4 m- and 6 m-long beams with a uniformly distributed load across the entire length. Sections of 350 and 500 mm (height), and 250 and 300 mm (width), were selected based on the longer beam. Beams were calculated to meet the required bending and shear strengths using bars and fibre reinforcements.

In both beams, the use of polyolefin fibres reduced the structural weight. SFRC20 behaved similarly to the use of stirrups, while SFRC35 was the least convenient of all the studied fibre reinforcements for moderate loads. A length increase of 50% from 4 m to 6 m means a load decrease of about 50% for the same reinforcement weight.

In terms of material and labour cost, PFRC10 incurs a higher cost for smaller loads, but the difference is reduced when loads increase. SFRC35 is the most expensive, and the cost is even higher for high loads due to the need of stirrups.

With respect to execution time, the SFRC35 option is the most efficient, followed by PFRC10. In all cases, FRC obtained better results than the bar-reinforced options. This result is even more visible when the design was focused on the shear strength only. For the same execution time, a 100% increment in the beam length (from 2 m to 4 m) means a load reduction of approximately 60%.

**Figure 16:**
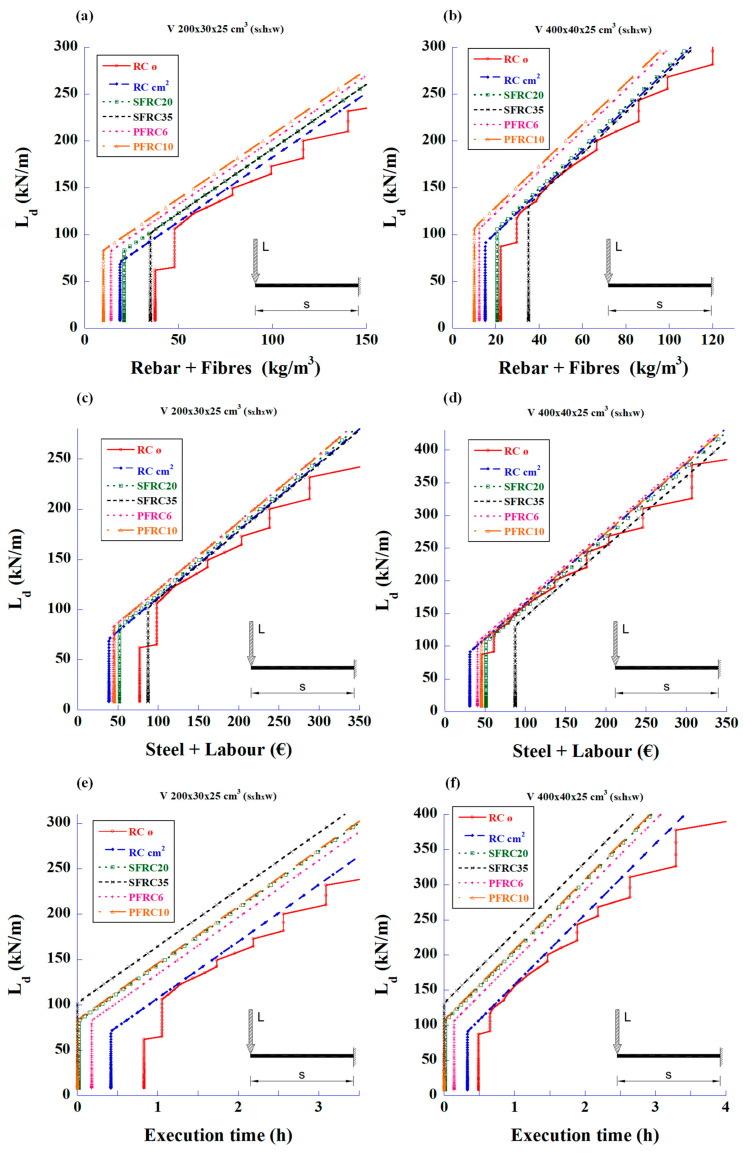
Cantilever beam with concentrated load designed for shear strength. Graphs relating design load vs. reinforcement weight; design load vs. cost of reinforcement including labour; and design load vs. execution time: (**a**) load–weight for 2 m-long beam, (**b**) load–weight for 4 m-long beam, (**c**) load–cost for 2 m-long beam, (**d**) load–cost for 4 m-long beam, (**e**) load–time for 2 m-long beam, and (**f**) load–time for 4 m-long beam.

In the design considering only the shear strength, 2 m- and 4 m-long cantilever beams with sections of 350 and 400 mm (depth) by 250 mm (width) were selected. The resulting graphs exploring the design load vs. material weight, cost, and execution time for different reinforcement options with fibres and bars are shown in Figure 16.

Considering Figure 16, it can be seen that reinforcement with polyolefin fibres is the most efficient in terms of reinforcement weight when designing for shear strength. It is worth noting that PFRC10 shows a lower weight than PFRC6 because the latter requires the presence of a minimum number of stirrups, according to Equation (3) of CE-21. Contrarily, the option SFRC35 always has worse results compared to the stirrup option.

In terms of cost, the results are quite similar for all types of reinforcement, with the exception of SFRC35, which is clearly worse.

The options SFRC35 and PFRC10 require less execution time. The options PFRC6 and SFRC20 require the presence of stirrups (CE-21), and that means a longer execution time.

### 4.2. Substitution of Stirrups Reinforcement

With an overall review of all the preceding graphs, it becomes evident that FRC is a good solution for replacing stirrup reinforcement. However, it can be observed that for higher amounts of fibres, the high costs mean that they are not recommended. Similarly, low amounts of polyolefin fibres (6 kg/m^3^) may be penalized by the need for stirrups.

PFRC10 proved to be the most efficient mix to provide shear strength with a smaller use of reinforcing weight. This kind of shear reinforcement complies with the requirements of code CE-21 for assuming structural strength contribution and fulfils the minimum amount of shear reinforcement. SFRC20 and PFRC6 have similar behaviours, although SFRC20 has a heavier weight but still minimizes the need of stirrups.

## 5. Conclusions

The results for the SFRC and PFRC demonstrate that fibre can substitute, to some extent, part of the longitudinal reinforcement needed to provide the required flexural strength. Additionally, the fibres can reduce or even eliminate the need for stirrups for shear strength, which results in savings in cost and execution time.

High fibre content values (50 or 70 kg/m^3^) provide a shorter execution time but increase the reinforcement weight and cost, reaching values similar to or higher than the ones for bar reinforcement. A moderate amount of fibres (20 to 30 kg/m^3^) is appropriate to reach a good balance between cost and execution time reduction.

The use of polyolefin fibres delivers an important weight reduction compared to RC and SFRC, providing the required structural reinforcement. In terms of shear strength and execution time savings, the most suitable option is to use an amount of polyolefin fibres that complies with the minimum in CE-21 to avoiding the need for stirrups, such as PFRC10.

Apart from the execution time and labour savings, an additional advantage of the use of fibres is the reduction in human errors and avoidance of potential danger in reinforcing work.

## Figures and Tables

**Table 1:** Beam types, dimensions, and fibre contents.

Typology	Span (mm)	Height (mm)	Width (mm)	SFRC (kg/m^3^)	PFRC (kg/m^3^)
**Simply Supported**					
Point load	4000	350	250	20, 35, 50 and 70	6 and 10
	6000	500	300	20, 35, 50 and 70	6 and 10
Uniformly distributed load	4000	350	250	20, 35, 50 and 70	6 and 10
	6000	500	300	20, 35, 50 and70	6 and 10
**Cantilever**					
Point load	2000	300	250	20, 35, 50 and 70	6 and 10
	4000	400	250	20, 35, 50 and 70	6 and 10
Uniformly distributed load	2000	300	250	20, 35, 50 and 70	6 and 10
	4000	400	250	20, 35, 50 and 70	6 and 10

**Table 2:** Main characteristics of the fibres.

Fibre	Dramix 3D	Maccaferri	Dramix OL	Dramix 5D	SikaFiber T-48
Material	Steel	Steel	Steel	Steel	Polyolefin
Length (mm)	50	50	13	60	48
Fibre shape	Hooked	Hooked	Straight	Hooked	Straight
Eq. diameter (mm)	1.05	1.00	0.20	0.90	0.90
Aspect ratio (*L*/ø)	45	50	65	65	53
Tensile strength (MPa)	1115	>1100	3050	2300	>400

**Table 3:** Mix dosage concrete (kg/m^3^) and the process of making concrete.

Material	Quantity	Process	Duration (s)
Cement CEM-I 52.5 R	375	Homogenization of aggregates	60
Limestone	200	Add 1/3 of fibres and mix	30
Water	188	Add cement and limestone powder	30
Water/cement	0.5	Add 1/3 of fibres and mix	30
Gravel 4–12	245	Add 75% of the mixing water and mix	30
Grit 4–8	367	Add 1/3 of the fibres and mix	30
Sand 0–2	918	Add 25% of the water with superplasticizer	240
Superplasticizer (% cement)	1.25	Rest (superplasticizer acting period)	150
		Final mix	120

**Table 4:** Unit prices for comparison between RC and FRC in beams subjected to shear forces and break down of the unit price of one kg of B500S steel bar reinforcement [8].

Unit	Unit Price ND Designation	Price (EUR/kg)
kg	Steel fibres Dramix^®^ RC65/35BN	2.47
kg	Polyolefin fibres SikaFiber^®^ T-48	4.54
kg	Steel rebars type B500S cut, bent, elaborated, and placed, including proportional part of trimming and tying of bars	2.12
**Decomposition of the price of iron**		
**Materials**		
Unit	Approved plastic spacer for reinforcing beams of various diameters	0.02
kg	Rebars made in an industrial workshop with steel in corrugated bars, UNE-EN 10,080 B500S, various diameters	1.60
kg	Galvanized binding wire, 1.30 mm diameter	0.02
	Subtotal materials	1.64
**Labour force**		
h	First officer iron worker	0.21
h	Assistant of first officer iron worker	0.22
	Subtotal labour force	0.43
**Direct cost**		
%	Direct costs	0.05
	Subtotal direct costs	0.05
**Total**		2.12

## Data Availability

The original contributions presented in the study are included in the article, further inquiries can be directed to the corresponding author.
